# Multiparametric MRI Assessment of Cervical Lymphadenopathy: Combined Diagnostic Performance of Morphological Features and Apparent Diffusion Coefficient

**DOI:** 10.3390/healthcare14111524

**Published:** 2026-05-30

**Authors:** Iulian-Alexandru Taciuc, Mihai Dumitru, Daniela Vrinceanu, Andreea Nicoleta Marinescu, Crenguta Serboiu, Adrian Costache, Adina Zamfir-Chiru-Anton

**Affiliations:** 1 Pathology Department, University of Medicine and Pharmacy “Carol Davila”, 050474 Bucharest, Romania; alexandertaciuc@gmail.com (I.-A.T.); adriancostacheeco@yahoo.com (A.C.); 2 ENT Department, University of Medicine and Pharmacy “Carol Davila”, 050474 Bucharest, Romania; daniela.vrinceanu@umfcd.ro; 3 Radiology Department, University of Medicine and Pharmacy “Carol Davila”, 050474 Bucharest, Romania; andreea_marinescu2003@yahoo.com (A.N.M.); crengutas@yahoo.com (C.S.); 4 ENT Department, Grigore Alexandrescu Children Hospital, 011743 Bucharest, Romania; zamfiradina@yahoo.com

**Keywords:** ADC, cervical lymphadenopathy, diffusion MRI, morphological metrics

## Abstract

**Highlights:**

This retrospective lesion-based study evaluated 88 cervical lymph nodes from 39 head-and-neck magnetic resonance imaging (MRI) examinations (September 2023–December 2025). Conventional MRI morphological features (such as shape, margins, hilum, necrosis, and long/short-axis ratio) were analyzed together with the apparent diffusion coefficient (ADC) values. Statistical analysis included non-parametric tests, ROC analysis, DeLong comparison, Firth penalized logistic regression for complete separation, generalized estimating equations to account for intra-patient clustering, patient-level bootstrap resampling, internal validation, and reproducibility assessment.

**What are the main findings?**

- Malignant lymph nodes had significantly lower ADC values than benign nodes. ADC alone achieved an AUC of 0.886, with an optimal cutoff of 0.900 × 10^−3^ mm^2^/s.
- A combined model including the ADC, nodal shape, and margin characteristics improved the diagnostic discrimination, with an apparent GEE AUC of 0.956, LOPO-CV AUC of 0.929, and optimism-corrected AUC of 0.949.
- DeLong testing confirmed that the combined model significantly outperformed ADC alone, with an AUC improvement of 0.070 (*p* = 0.006).
- The inter- and intra-observer reproducibility was excellent for ADC measurements and categorical morphological features.

**What are the implications of the main findings?**

- A structured multiparametric MRI assessment combining diffusion-derived ADC values with qualitative morphological features may improve cervical lymph node stratification compared with ADC alone.
- The findings support the use of standardized MRI reporting elements, including nodal shape, margin characteristics, fatty hilum status, necrosis, and ADC measurement, while recognizing that external validation in larger multicenter cohorts is required before routine clinical implementation can be recommended.

**Abstract:**

**Background:** Accurate differentiation between benign and malignant cervical lymphadenopathy remains clinically important for diagnostic stratification and treatment planning. This study evaluated the diagnostic performance of conventional morphological magnetic resonance imaging (MRI) features and apparent diffusion coefficient (ADC) values in a mixed cohort of cervical lymphadenopathies. **Methods:** This retrospective lesion-based diagnostic study included 88 cervical lymph nodes from 39 patients who underwent head-and-neck MRI between September 2023 and December 2025. The cohort had malignant entities such as squamous cell carcinoma metastases, thyroid carcinoma, non-Hodgkin lymphoma, adenoid cystic carcinoma, and medullary thyroid carcinoma, as well as benign/reactive, inflammatory, CMV-related, tuberculous, Warthin tumor-associated, and cystic lymphangioma-related lymphadenopathies. MRI examinations were performed for heterogeneous indications, including the initial assessment of palpable cervical lymphadenopathy, oncological staging, post-biopsy follow-up, suspected recurrence, and benign/inflammatory lesion characterization; therefore, not all patients underwent MRI for the same clinical indication. Most examinations were performed during the initial diagnostic work-up, while six cases represented post-biopsy follow-up. Morphological features and ADC values were analyzed using Mann–Whitney U tests, chi-square tests, ROC analysis, DeLong testing, Firth penalized logistic regression, generalized estimating equations (GEE), patient-level bootstrap resampling, and calibration analysis. Statistical analyses were performed using Python (Version 3.12), with exploratory verification in JASP. Statistical significance was set at *p* < 0.05. **Results:** The cohort included 39 patients with a mean age of 54 years (range: 18–74 years), with 20 males and 19 females. Of the 88 lymph nodes, 33 were malignant and 55 benign. Malignant nodes demonstrated significantly lower ADC values than benign nodes (0.87 ± 0.23 vs. 1.25 ± 0.22 × 10^−3^ mm^2^/s; U = 207, *p* < 0.001). ADC alone showed good diagnostic performance, with an AUC of 0.886 (95% CI: 0.803–0.960). The optimal ADC cutoff was 0.900 × 10^−3^ mm^2^/s, yielding 75.8% sensitivity and 89.1% specificity. The final GEE model, including the ADC, nodal shape, and margin characteristics while accounting for intra-patient clustering, achieved an apparent AUC of 0.956. Leave-one-patient-out cross-validation yielded an AUC of 0.929, and the bootstrap optimism-corrected AUC was 0.949. DeLong testing confirmed that the combined model significantly outperformed the ADC alone (AUC improvement = 0.070; *p* = 0.006), and the inter- and intra-observer reproducibility for ADC was excellent. **Conclusions:** ADC values, nodal shape, and margin characteristics provide complementary diagnostic information for differentiating benign from malignant cervical lymph nodes. A structured multiparametric MRI approach demonstrated high diagnostic performance, although the findings should be interpreted in the context of the retrospective single-center design and histopathological heterogeneity.

## 1. Introduction

The head-and-neck region contains more than 300 lymph nodes, which constitute a critical component of the immune system and play an essential role in immune surveillance and response [1,2]. The majority of inflammatory or infectious conditions involving cervical lymph nodes are typically assessed through clinical examination and laboratory investigations and, therefore, do not routinely require imaging studies. However, imaging becomes particularly important in the evaluation of head-and-neck malignancies, especially for the detection and characterization of metastatic lymph node involvement [3]. Postoperative histopathological examination remains the gold standard for the detection and confirmation of metastatic involvement in cervical lymphadenopathy. It provides definitive diagnostic information regarding the presence, extent, and characteristics of nodal metastases, thereby serving as the reference standard against which clinical and imaging findings are compared [4].

There has been increasing emphasis on the prompt and accurate differentiation between benign and malignant lymphadenopathies. Early identification of malignant nodal disease is essential, as it significantly influences the staging, therapeutic decision-making, and overall prognosis [1,2].

Cervical lymph node metastases most commonly arise from primary malignancies of the head-and-neck region, particularly squamous cell carcinomas originating from the oral cavity, oropharynx, hypopharynx, and larynx, which represent the predominant histological subtype in this anatomical area [5,6]. Thyroid carcinoma, especially papillary thyroid carcinoma, frequently metastasizes to central and lateral cervical nodal compartments [7]. Salivary gland malignancies, including mucoepidermoid carcinoma and adenoid cystic carcinoma, may also demonstrate regional nodal spread depending on the tumor grade and stage [6]. In addition, nasopharyngeal carcinoma is characteristically associated with early and often bilateral cervical lymph node involvement [8].

Current medical practice offers multiple imaging modalities for the evaluation of cervical lymphadenopathy, including ultrasonography (US), computed tomography (CT), and MRI, each with distinct strengths and limitations. In our previous work, we detailed the advantages of US and successfully implemented a simple convolutional neural network to improve the identification of malignant lymphadenopathy [9]. In contrast, MRI-based diagnostic strategies for lymph node evaluation have undergone relatively limited conceptual evolution in recent years, despite rapid technological advancements. Furthermore, the absence of standardized MRI criteria for characterizing cervical lymphadenopathy contributes to variability in diagnostic performance and interobserver agreement [3].

However, the identification of lymph node metastases on magnetic resonance imaging (MRI) in the absence of overt signs such as extranodal extension (ENE) or intranodal necrosis remains challenging. In these cases, conventional diagnostic criteria based primarily on nodal size and morphological features are often insufficiently sensitive and specific, limiting their reliability in distinguishing benign from malignant lymphadenopathy [10].

Among advanced imaging modalities, hybrid [18F]FDG PET/MRI has shown particularly high diagnostic accuracy in head-and-neck squamous cell carcinoma by combining metabolic information with the superior soft-tissue contrast of MRI. Recent systematic review-based and meta-analytic evidence supports the value of this modality for primary tumor assessment and nodal staging. However, PET/MRI availability remains limited, and conventional MRI with DWI continues to represent a more accessible and widely implemented diagnostic approach. Accordingly, further refinement of MRI-based morphological and diffusion criteria remains clinically relevant [11].

Diffusion-weighted imaging (DWI) is a non-invasive functional MRI technique that characterizes tissues according to the degree of restriction in the diffusion of water molecules within the extracellular and intracellular spaces. As a relatively simple and rapid sequence, DWI is routinely incorporated into standard head-and-neck MRI protocols [12]. Quantitative analysis of diffusion restriction is achieved through the calculation of apparent diffusion coefficient (ADC) values. Malignant lymph nodes typically demonstrate lower ADC values compared with benign or reactive nodes, reflecting higher cellular density and reduced extracellular space, which impede the movement of water molecules. Therefore, ADC measurements may provide additional diagnostic value in improving the accuracy of nodal characterization, particularly in cases where conventional morphological criteria are inconclusive [4,13].

Several morphological features of cervical lymph nodes identified on MRI have been associated with metastatic involvement. These include a short-axis diameter larger than 10 mm, a rounded nodal configuration, the presence of central necrosis, evidence of extracapsular tumor spread (also referred to as extranodal extension), and regional clustering of lymph nodes. However, diagnostic assessment becomes considerably more challenging in lymph nodes that are normal in size or lack necrotic changes [14].

Although both morphological MRI criteria and diffusion-derived ADC values have been individually reported as useful markers for nodal characterization, their relative contribution and incremental diagnostic value when combined remain incompletely defined. Furthermore, the independent role of conventional axial diameter ratios in the presence of qualitative three-dimensional morphological assessment has not been consistently evaluated.

This study aimed to assess the independent predictive value of conventional morphological MRI features and ADC measurements in cervical lymph node characterization, evaluate the diagnostic contribution of the axial long-to-short diameter ratio after adjustment for qualitative morphological features, and determine the incremental performance of a combined morphologic–diffusion model. To our knowledge, few studies have systematically evaluated the independent and combined contribution of qualitative morphology and quantitative diffusion parameters using multivariate modeling with the assessment of collinearity and incremental ROC performance. Although ADC values and conventional morphological MRI features have been individually studied in cervical lymph node assessment, their combined contribution in heterogeneous real-world cohorts remains clinically relevant, particularly when multiple lymph nodes are assessed per patient. In this context, the added value of a multiparametric model that integrates ADC with qualitative three-dimensional morphological assessment, while accounting for intra-patient clustering and assessing internal validation, remains insufficiently characterized. Therefore, this study aimed to evaluate the independent and combined diagnostic performance of ADC values and conventional MRI morphological features for differentiating benign from malignant cervical lymph nodes using a lesion-based analysis with patient-level clustering adjustment.

## 2. Materials and Methods

### 2.1. Database

**Figure 1:**
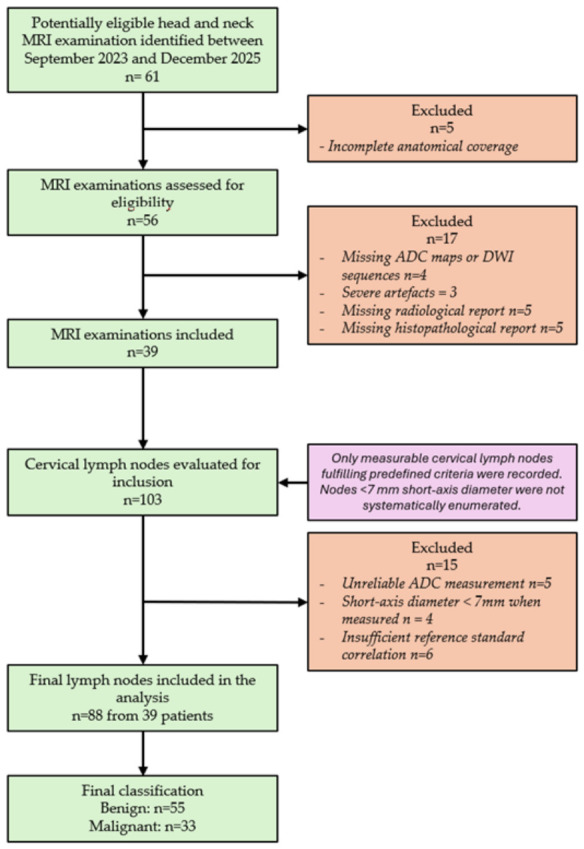
STARD-compliant patient and lymph-node flow diagram. Flow diagram showing the selection of MRI examinations and cervical lymph nodes included in the diagnostic analysis. The final cohort included 88 measurable cervical lymph nodes from 39 patients. Lymph nodes under the predefined short-axis threshold of 7 mm were not systematically enumerated during retrospective data extraction.

This small, retrospective, exploratory study included 88 cervical lymph nodes identified in 39 MRI examinations. The lymph nodes were evaluated individually and correlated with the histopathological findings. The participants formed a retrospective convenience series of eligible head-and-neck MRI examinations performed between September 2023 and December 2025 (Figure 1).

MRI examinations were performed for heterogeneous clinical indications related to cervical lymphadenopathy and head-and-neck pathology. Indications included the initial assessment of palpable cervical lymphadenopathy, preoperative staging of suspected or confirmed head-and-neck malignancy, evaluation of suspected nodal recurrence, post-biopsy follow-up, and characterization of inflammatory or benign neck lesions.

The inclusion criteria were as follows: (1) clinical examination demonstrating palpable cervical lymphadenopathy; (2) MRI evaluation of the head-and-neck region; (3) availability of both MRI images and the corresponding radiological report; (4) MRI protocol including at minimum T2-weighted fat-suppressed sequences and diffusion-weighted imaging (DWI); (5) histopathological confirmation of either the primary tumor or the lymphadenopathy; (6) measurable short-axis diameter of at least 7 mm, with no upper size threshold (therefore, both borderline-sized and enlarged lymph nodes were included).

If the histopathological analysis of the primary tumor demonstrated benign pathology, the associated lymphadenopathies were not subjected to additional biopsy. In cases where no primary tumor was identified, suspicious lymph nodes were directly biopsied for diagnostic confirmation. In patients who underwent surgical resection of the primary tumor with lymphadenectomy, pathological findings were retrieved from the surgical specimen reports.

The exclusion criteria included major motion or susceptibility artifacts, non-palpable lymph nodes, incomplete imaging or missing radiological reports, and lymph nodes measuring less than 7 mm in short-axis diameter. Lymph nodes with a short-axis diameter less than 7 mm were excluded because of reduced measurement reliability, increased partial-volume effects, and limited confidence in the ADC ROI placement.

Of the 88 lymph nodes analyzed, 33 (37.5%) were malignant, and 55 (62.5%) were benign. Malignant nodes were primarily associated with squamous cell carcinomas and non-Hodgkin lymphoma, while benign nodes were predominantly reactive/inflammatory in origin. Multiple lymph nodes were derived from some patients, which may introduce clustering effects.

For oncological cases, available TNM staging information was retrieved from the clinical and histopathological records when present. Nodal staging was considered according to the TNM/AJCC framework applicable to the corresponding primary tumor type. However, because this study was designed as a lymph-node-level diagnostic performance analysis and included both malignant and benign/inflammatory entities, formal patient-level N staging was not used as an endpoint in the statistical analysis. The primary reference outcome was binary benign or malignant lymph node classification.

### 2.2. MRI Protocol

**Figure 2:**
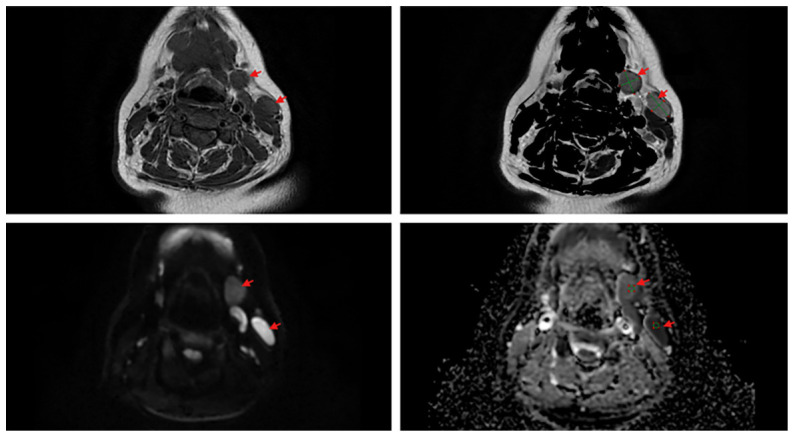
Example of ADC measurement. ADC was measured using a manually placed representative ROI within the solid component of the lymph node, avoiding adjacent vessels, fat, necrotic/cystic areas, and partial-volume artifacts. This figure illustrates the ROI placement strategy used for ADC extraction.

MRI examinations were performed using a 3.0 Tesla system (Toshiba Medical Systems, Otawara, Japan) within the same healthcare institution, using a dedicated neck phased-array coil. Patients were examined in the supine position. The imaging protocol included axial and coronal T1-weighted, T2-weighted, and fat-suppressed T2-weighted sequences, followed by diffusion-weighted imaging (DWI). Contrast-enhanced sequences were also acquired. The slice thickness ranged from 3 to 5 mm depending on the sequence and imaging plane. Diffusion-weighted imaging was performed using a single-shot echo-planar imaging sequence. Across the cohort, the low-b-value reference was b = 50 s/mm^2^, while the high-b-value diffusion weighting was either b = 800 or b = 1000 s/mm^2^, depending on the acquisition protocol. No true b = 0 image was consistently available in the analyzed DICOM data. ADC measurements were performed on vendor-generated ADC maps using manually placed circular or oval ROIs. ROIs were positioned within the visually most representative solid component of each lymph node, avoiding necrotic or cystic areas, visible vessels, adjacent fat, and partial-volume artifacts (Figure 2). Whole-node segmentation was not performed. The same ROI placement strategy was applied consistently across all cases.

### 2.3. Image Analysis

Image analysis was performed retrospectively on the institutional PACS workstation. Morphological and diffusion parameters were evaluated for each lymph node included in the study.

At the time of the original clinical MRI interpretation, radiologists were blinded to the final histopathological diagnosis in 33 cases, as imaging was performed during the initial diagnostic work-up. In six cases, MRI was performed as post-biopsy follow-up, and the pathological diagnosis was available in the clinical imaging report. For the present retrospective analysis, the research team had access to both the imaging reports and the histopathological findings; therefore, complete blinding during imaging feature extraction and ADC measurement was not ensured.

Morphological assessment was conducted using conventional MRI sequences (T1-weighted, T2-weighted, and T2 fat-suppressed images). The lymph node shape was classified as oval (0) or round (1) based on the global three-dimensional appearance rather than a solely axial configuration. The margins were categorized as smooth (0) or irregular/lobulated (1). The presence of central necrosis was recorded when areas of high T2 signal intensity and corresponding low signal on T1-weighted images were identified within the nodal parenchyma. The fatty hilum was considered preserved (1) when a central fatty signal was clearly identifiable and absent (0) otherwise. The axial long-to-short diameter ratio (L/l) was calculated on the axial images by measuring the maximal longitudinal and perpendicular short-axis diameters of each lymph node.

For diffusion analysis, ADC measurements were performed on automatically generated ADC maps. Circular regions of interest (ROI) were manually placed within the solid portion of each lymph node, carefully avoiding necrotic or cystic areas and excluding adjacent fat or vessels. In cases of heterogeneous signal intensity, the ROI was positioned in the most representative solid component. ADC ROI placement was performed using the RadiAnt DICOM Viewer. Inter-observer analysis was performed independently using the Carestream Vue PACS software 12.0.

### 2.4. Collected Variables and Statistical Software

For each patient, demographic and clinical variables were collected, including age, sex, clinical indication for MRI, primary histopathological diagnosis, and available oncological staging information when applicable. For each lymph node, the following imaging variables were recorded: anatomical location, laterality, short-axis diameter, long-axis diameter, long-to-short axis ratio, nodal shape, margin characteristics, presence or absence of fatty hilum, presence or absence of necrosis, diffusion restriction, and ADC value.

The nodal shape was coded as oval or round, the margins as smooth or irregular/lobulated, the fatty hilum as absent or preserved, and necrosis as absent or present. The ADC values were recorded as continuous variables and expressed in ×10^−3^ mm^2^/s. The final reference outcome was the binary lymph node status, classified as benign or malignant according to the available histopathological reference standard and clinical–pathological correlation.

Data organization and preprocessing were performed in Microsoft Excel. Statistical analyses were performed using Python, including the pandas, numpy, scipy, statsmodels, scikit-learn, and matplotlib libraries. Initial descriptive analyses and contingency table exploration were also verified using the JASP software (version 0.95.4). Generalized estimating equations, bootstrap resampling, ROC analysis, DeLong testing, calibration assessment, and internal validation were performed in Python.

### 2.5. Reference Standard

The reference standard was based on the histopathological and clinicopathological data. When available, direct nodal histopathology from biopsy, excision, or lymphadenectomy specimens was used to classify lymph nodes as benign or malignant. In patients undergoing surgical treatment with lymphadenectomy, nodal status was extracted from the final pathology report. In cases in which lymph nodes were not individually biopsied, nodal classification was based on the histopathological diagnosis of the primary lesion together with clinicoradiological correlation, including the radiological report and clinical follow-up information when available.

Lymph nodes were classified as malignant when metastatic involvement or lymphomatous infiltration was confirmed histopathologically. Lymph nodes were classified as benign when nodal histopathology demonstrated reactive, inflammatory, infectious, or benign pathology, or when associated with a histopathologically confirmed benign primary lesion, and no malignant nodal involvement was documented. The final reference outcome used for statistical analysis was binary lymph node status: benign or malignant.

### 2.6. Statistical Analysis

The primary analysis was performed at the lymph-node level, with each lymph node considered as an individual diagnostic unit. Therefore, the study represents a lesion-based diagnostic performance analysis rather than a patient-based staging analysis.

Comparisons between benign and malignant lymph nodes were performed using the chi-square test for categorical variables and the Mann–Whitney U test for non-normally distributed continuous variables. Effect sizes were reported as odds ratios (OR) with 95% confidence intervals for categorical predictors and rank-biserial correlation coefficients for non-parametric comparisons.

To assess the potential influence of diffusion weighting on the ADC measurements, the ADC values obtained at b = 800 and b = 1000 s/mm^2^ were compared. As no statistically significant difference was observed between acquisition modes (median 1.05 vs. 1.15 × 10^−3^ mm^2^/s, U = 1055, *p* = 0.134), the ADC values were subsequently pooled for further analyses in order to increase the statistical power and ensure stable ROC estimation.

Variables demonstrating statistical significance in univariate analysis were entered into multivariate logistic regression models to identify independent predictors of malignancy. Multicollinearity was evaluated using variance inflation factors (VIF), with values below 5 considered acceptable.

The diagnostic performance was assessed using receiver operating characteristic (ROC) curve analysis. The area under the curve (AUC), sensitivity, specificity, and overall accuracy were calculated. Statistical significance was defined as *p* < 0.05.

To account for intra-patient clustering due to multiple lymph nodes per patient, generalized estimating equations (GEE) with exchangeable correlation structures were applied. Robust Wald confidence intervals and patient-level cluster bootstrap confidence intervals (1000 resamples) were calculated. Unstable bootstrap replicates were excluded from the final bootstrap estimation.

Reproducibility analysis was performed on a subset of 41 lymph nodes from 17 patients. For inter-observer reproducibility, a second radiologist independently evaluated the same lymph nodes using the predefined imaging criteria. For intra-observer reproducibility, the primary reader repeated the ADC measurements and categorical morphological assessment after a washout interval, blinded to the initial measurements. ADC reproducibility was assessed using the intraclass correlation coefficient for absolute agreement, while categorical variables, including the nodal shape, margin characteristics, fatty hilum status, and necrosis, were assessed using Cohen’s kappa. The analyzed subset included both benign and malignant lymph nodes.

Finally, because of the retrospective nature of the study, no formal a priori sample-size calculation was performed. A post hoc sample-size justification was therefore conducted based on the number of malignant events available for multivariable modeling. The final GEE model included three predictors (ADC, nodal shape, and margin characteristics) and 33 malignant lymph nodes, corresponding to approximately 11 malignant events per predictor. This was considered acceptable for an exploratory diagnostic model while maintaining a parsimonious predictor set to reduce the risk of overfitting.

## 3. Results

### 3.1. Demographic Characteristics and Histopathological Profile of the Cohort

**Figure 3:**
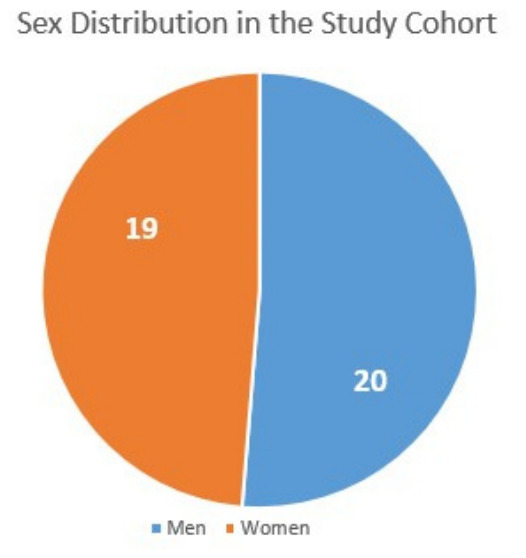
The sex distribution in the study group.

**Figure 4:**
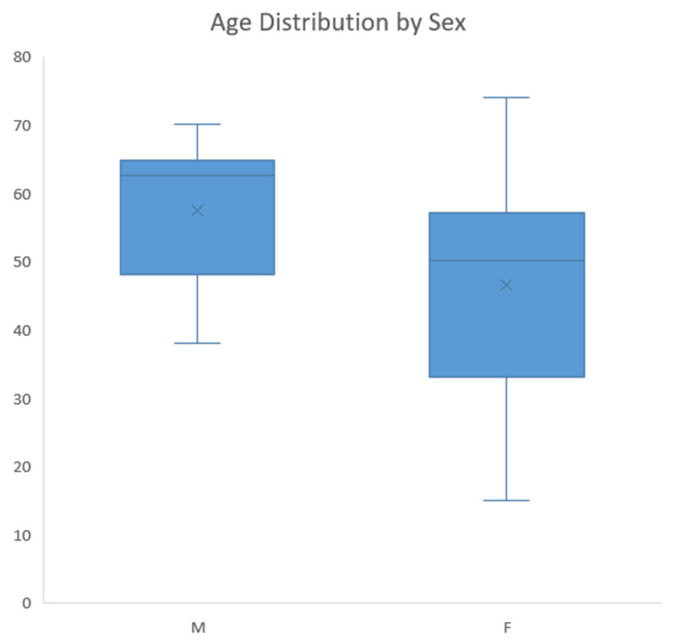
The study group distribution by age and sex.

The study population included 39 patients with an almost equal sex distribution (Figure 3) and a mean age of 54 years (range: 18–74 years) (Figure 4). MRI examinations were performed in heterogeneous clinical contexts, including an initial evaluation of cervical lymphadenopathy, assessment of suspected or confirmed head-and-neck malignancy, post-biopsy follow-up, and characterization of benign or inflammatory lesions. However, standardized pre- and post-MRI management plans were not consistently available in the retrospective records; therefore, a formal quantitative analysis of MRI-driven management changes was not performed.

A histopathological breakdown of the 88 lymph nodes is provided in Table 1. Overall, 33 lymph nodes were classified as malignant and 55 as benign. Malignant lymph nodes were most frequently associated with squamous cell carcinoma, followed by papillary thyroid carcinoma, non-Hodgkin lymphoma, adenoid cystic carcinoma, and medullary thyroid carcinoma. Benign lymph nodes were predominantly reactive/inflammatory, with additional benign entities including CMV lymphadenitis, tuberculous lymphadenitis, Warthin tumor-associated reactive nodes, and cystic lymphangioma. Because several histopathological categories contained only a small number of patients or lymph nodes, formal subgroup analysis according to individual histopathological diagnosis was not performed. The histopathology was used primarily as the reference standard for the binary classification of lymph nodes as benign or malignant.

### 3.2. Morpho-Imaging Parameters Analysis

**Figure 5:**
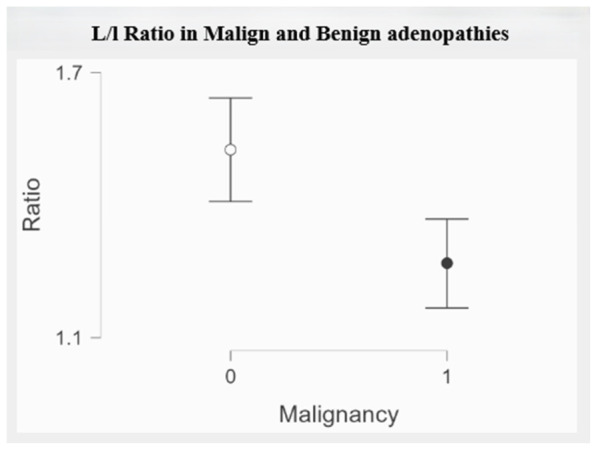
The analysis of lymph node dimensions on MRI.

The ratio of axial diameters was analyzed using the Mann–Whitney U test. The axial L/l ratio was significantly lower in malignant compared to benign lymph nodes (U = 1283.5, *p* = 0.001). Malignant nodes demonstrated a more rounded configuration (mean 1.268 ± 0.285) compared to benign nodes (mean 1.526 ± 0.432) (Figure 5). The effect size indicated a moderate-to-large association (rank-biserial r = 0.414, 95% CI: 0.188–0.599). Receiver operating characteristic (ROC) analysis demonstrated moderate discriminatory ability (AUC = 0.70), with a sensitivity of 40% and a specificity of 70% at the selected cutoff value.

The lymph node shape demonstrated a statistically significant association with malignancy (χ^2^ = 32.26, *p* < 0.001). Rounded lymph nodes were significantly more likely to be malignant compared to oval nodes (OR = 18.2, 95% CI: 6.0–54.6). The distinction between nodal shape and the L/l ratio lies in the fact that the shape reflects the three-dimensional configuration of the lymph node, whereas the L/l ratio represents a two-dimensional axial measurement.

The lymph node margins were significantly associated with malignancy (χ^2^ = 20.85, *p* < 0.001). Nodes presenting lobulated or irregular margins had a markedly increased likelihood of being malignant compared to those with smooth margins (OR = 19.5, 95% CI: 4.0–94.0). Lymph node necrosis was significantly associated with malignancy (χ^2^ = 7.54, *p* = 0.006). Nodes presenting necrotic features demonstrated an increased likelihood of malignancy (OR = 12.0, 95% CI: 1.3–104.7). The wide confidence interval may reflect the low number of necrotic benign cases, including one instance of cystic lymphangitis.

Preservation of the lymph node hilum was strongly associated with benignity (χ^2^ = 72.7, *p* < 0.001). No malignant lymph nodes demonstrated a preserved hilum. The presence of a hilum was associated with a markedly reduced likelihood of malignancy (OR = 0.001, 95% CI: 0.0001–0.026). The contingency data, odds ratios, 95% confidence intervals, and *p*-values are presented in Table 2.

In the multivariate logistic regression analysis, the lymph node shape (OR ≈ 22.7, *p* < 0.001) and irregular margins (OR ≈ 20.7, *p* = 0.002) remained independent predictors of malignancy. The axial L/l ratio and necrosis lost statistical significance after adjustment. Multicollinearity was assessed using variance inflation factors (VIF), which ranged from 1.07 to 1.69, confirming the absence of significant collinearity among independent variables. Because a preserved fatty hilum produced complete separation in conventional logistic regression, its association with malignancy was assessed using Firth’s penalized logistic regression. A preserved fatty hilum was strongly associated with a reduced likelihood of malignancy (OR = 0.001, 95% CI: 0.00007–0.026, *p* < 0.001). Due to complete separation and model stability considerations, the fatty hilum was excluded from the final multivariable GEE model.

### 3.3. ADC Implications

To ensure that differences in the diffusion weighting (b = 800 vs. 1000 s/mm^2^) did not introduce bias or instability in the ROC analysis, ADC values obtained with the two protocols were compared. No statistically significant difference was observed between ADC values measured at b = 800 and b = 1000 s/mm^2^ (median 1.05 vs. 1.15 × 10^−3^ mm^2^/s, U = 1055, *p* = 0.134).

Given the absence of significant differences, the ADC values were subsequently analyzed collectively, regardless of the applied diffusion weighting.

**Figure 6:**
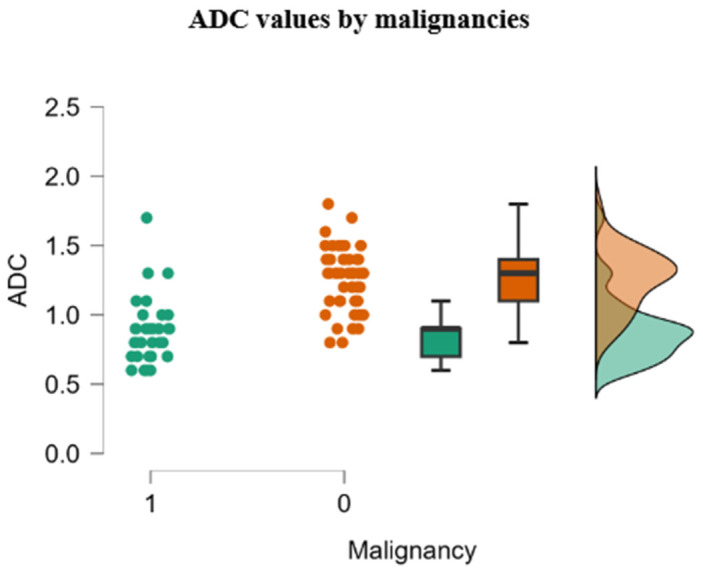
Distribution of ADC values according to lymph node malignancy status. Malignant lymph nodes showed significantly lower ADC values than benign nodes (0.87 ± 0.23 vs. 1.25 ± 0.22 × 10^−3^ mm^2^/s; *p* < 0.001).

The ADC values were significantly lower in malignant compared to benign lymph nodes (U = 207, *p* < 0.001) (Figure 6). Malignant nodes demonstrated a mean ADC of 0.87 ± 0.23 × 10^−3^ mm^2^/s, whereas benign nodes showed a mean ADC of 1.25 ± 0.22 × 10^−3^ mm^2^/s. The effect size indicated a very strong association (rank-biserial r = 0.772, 95% CI: 0.65–0.85).

**Figure 7:**
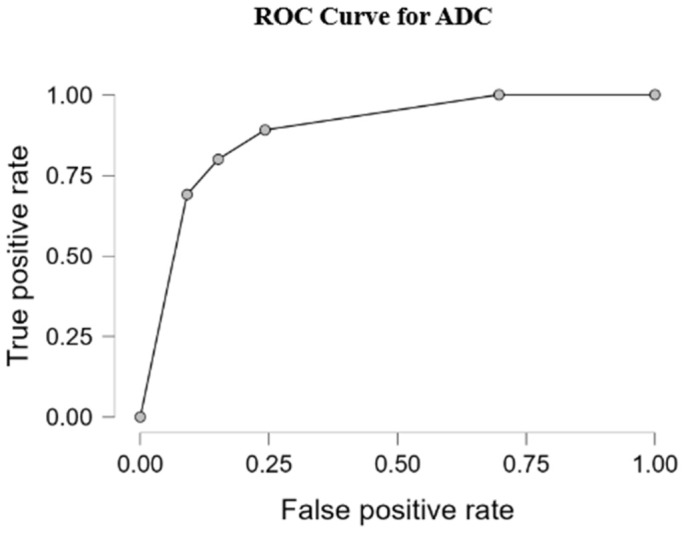
ROC curve for univariate analysis of ADC. ADC demonstrated an AUC of 0.886 (95% CI: 0.803–0.960). The optimal cutoff was 0.900 × 10^−3^ mm^2^/s, with a sensitivity of 75.8% and a specificity of 89.1%.

The standalone diagnostic performance of ADC was assessed using ROC analysis (Figure 7). Because lower ADC values were associated with malignancy, ADC values were directionally transformed for ROC estimation. ADC alone demonstrated good discriminative performance, with an AUC of 0.886 (95% CI: 0.803–0.960). The optimal threshold determined by the Youden index was 0.900 × 10^−3^ mm^2^/s, with values below this cutoff considered suggestive of malignancy. This threshold yielded a sensitivity of 75.8% and a specificity of 89.1%. Patient-level bootstrap resampling confirmed the stability of the ADC threshold, with a cutoff 95% CI of 0.900–1.100 × 10^−3^ mm^2^/s.

To account for intra-patient clustering resulting from the inclusion of multiple lymph nodes from the same patient, generalized estimating equations (GEE) with a binomial distribution and exchangeable correlation structure were applied for multivariable analysis. Variables exhibiting complete or quasi-complete separation (fatty hilum and necrosis) were excluded from the final multivariable model to ensure model stability. In addition, the nodal shape was retained instead of the long-to-short axis ratio, as it more comprehensively reflects three-dimensional nodal morphology while avoiding potential collinearity between morphologic parameters.

**Figure 8:**
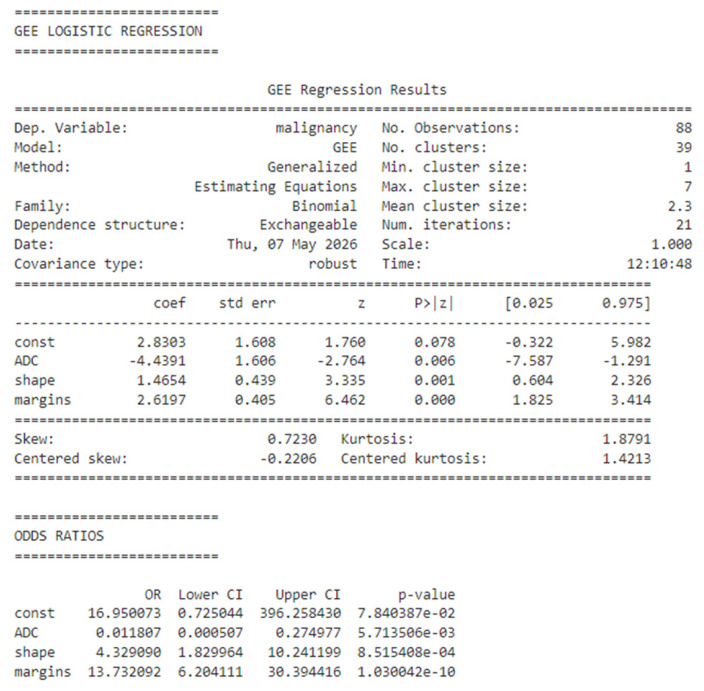
GEE regression results, generated in Python.

**Figure 9:**
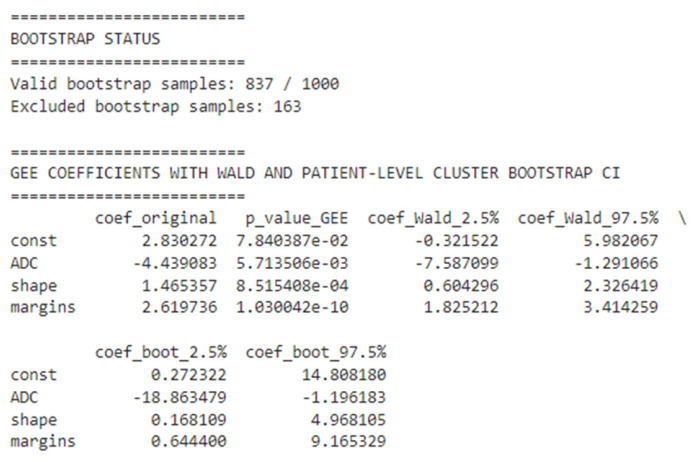
Bootstrap status.

In the final GEE model, the ADC values, nodal shape, and margin characteristics remained independent predictors of malignancy (Figure 8 and Figure 9). Lower ADC values were significantly associated with malignant lymph nodes (coefficient = −4.44, *p* = 0.006). Round nodal configuration was independently associated with malignancy (coefficient = 1.47, *p* = 0.001), while irregular margins demonstrated the strongest association with malignant involvement (coefficient = 2.62, *p* < 0.001). Robust Wald confidence intervals and patient-level cluster bootstrap confidence intervals were additionally calculated using 1000 bootstrap resamples. The bootstrap analysis demonstrated stable coefficient estimates across resamples, supporting the robustness of the final model.

**Figure 10:**
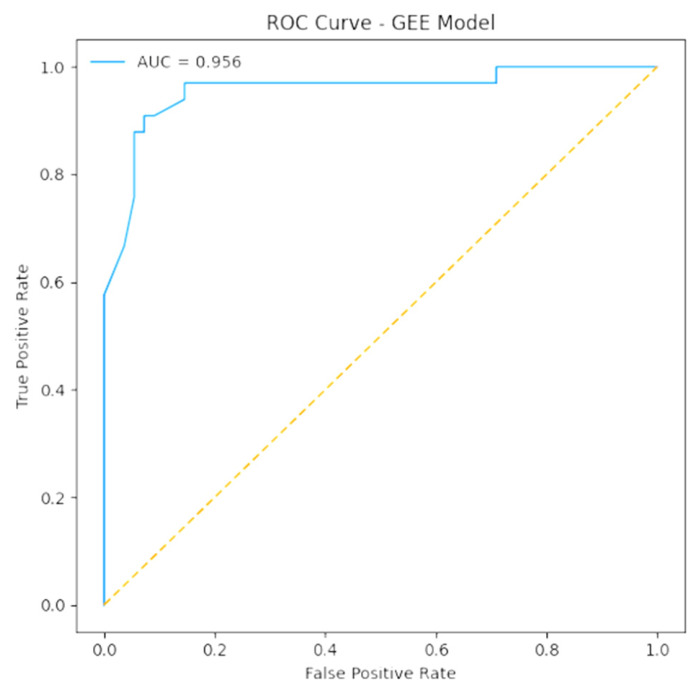
ROC curve for the multivariate analysis of ADC, which included the shape, margins, and ADC.

The apparent diagnostic performance of the final GEE model demonstrated an area under the ROC curve (AUC) of 0.956 (Figure 10). Internal validation using leave-one-patient-out cross-validation yielded a cross-validated AUC of 0.929, indicating good model stability and limited evidence of overfitting. Patient-level bootstrap optimism correction demonstrated a mean optimism of 0.006, resulting in an optimism-corrected AUC of 0.949. Calibration analysis demonstrated a calibration intercept of 0.380 and a calibration slope of 1.541, with variability across risk strata consistent with the limited sample size.

Pairwise comparison of ROC curves using DeLong’s test demonstrated that the combined GEE model significantly outperformed ADC alone, with an AUC improvement of 0.070 (AUC 0.956 vs. 0.886; z = 2.77, *p* = 0.006).

**Figure 11:**
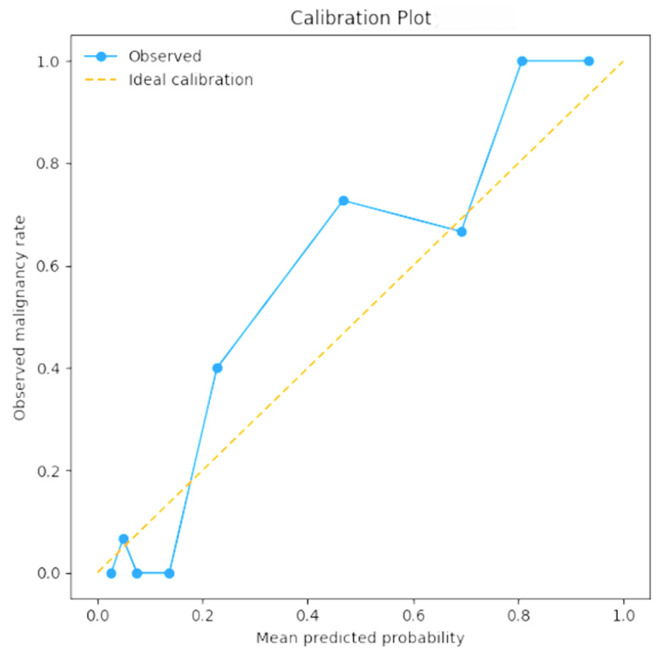
Calibration plot of the final GEE model. The *x*-axis represents the mean predicted probability of malignancy, and the *y*-axis represents the observed malignancy rate. The dashed diagonal line indicates ideal calibration. The calibration plot demonstrated acceptable overall agreement between the predicted and observed probabilities, with variability across risk strata likely related to the limited sample size.

Internal validation was additionally performed using patient-level bootstrap resampling with 1000 resamples. A total of 837 bootstrap resamples yielded stable model estimates and were retained for optimism correction. The apparent AUC of the final GEE model was 0.956, while the mean estimated optimism was 0.006, resulting in an optimism-corrected AUC of 0.949, suggesting limited optimism in the apparent model performance. Calibration was assessed using the calibration intercept, calibration slope, and a calibration plot (Figure 11). Calibration analysis demonstrated a calibration intercept of 0.380 and a calibration slope of 1.541. The calibration plot showed acceptable overall agreement between the predicted and observed probabilities, although variability was observed across risk strata, consistent with the limited sample size.

The reproducibility analysis demonstrated excellent agreement for ADC measurements. The inter-observer agreement was excellent, with ICC(A,1) = 0.963 (95% CI: 0.93–0.98, *p* < 0.001). The intra-observer agreement was also excellent, with ICC(A,1) = 0.983 (95% CI: 0.97–0.99, *p* < 0.001). For categorical morphological variables, the inter- and intra-observer agreement was perfect, with Cohen’s kappa values of 1.000 for the nodal shape, margin characteristics, fatty hilum status, and necrosis. The inter- and intra-observer reproducibility analyses were performed six weeks after the initial measurements.

Because of the retrospective design, adverse event reporting was limited to the information available in the medical records. No adverse events related to the MRI index test or the histopathological reference standard were documented in the reviewed records.

## 4. Discussion

The present study demonstrates that integrating morphological MRI features with diffusion-derived ADC values yields excellent diagnostic performance in differentiating malignant from benign cervical lymph nodes. While both morphology and ADC independently showed strong associations with malignancy, their combination significantly enhanced the discriminative ability, achieving an AUC of 0.956. These findings support a structured multiparametric MRI approach for nodal characterization.

Among morphological variables, the nodal shape and margin characteristics emerged as independent predictors of malignancy. A rounded configuration and irregular margins were strongly associated with malignant involvement, likely reflecting architectural distortion and capsular infiltration. In contrast, the axial long-to-short diameter ratio (L/l), although significant in univariate analysis, did not retain significance after multivariate adjustment. This suggests that a global morphological assessment may better capture structural alterations than isolated two-dimensional axial measurements.

The ADC values were significantly lower in malignant lymph nodes, consistent with increased cellular density and reduced extracellular diffusion space. The standalone diagnostic performance of ADC was very good (AUC = 0.886), confirming its role as a microstructural biomarker of malignancy. However, the substantial improvement observed when ADC was combined with morphological parameters highlights the incremental value of quantitative diffusion imaging beyond conventional structural assessment.

The present findings should be interpreted in the context of an already established body of literature supporting the role of ADC and morphological MRI features in cervical lymph node characterization. Accordingly, the contribution of this study is not the identification of ADC or morphology as new diagnostic markers but rather the evaluation of their combined performance within a structured multiparametric framework. Importantly, the revised analysis accounted for intra-patient clustering, included internal validation, compared ADC alone with the combined model using paired ROC analysis, and assessed the measurement reproducibility. These methodological additions strengthen the interpretation of the model performance and provide a more conservative estimate of the incremental diagnostic value of combining diffusion-derived and morphological MRI parameters. However, the model cannot yet be considered ready for routine clinical implementation without additional blinded testing and external validation.

The ADC measurements were obtained using manually placed representative ROIs rather than whole-node or volumetric segmentation. Although this approach reflects common clinical practice and allows the exclusion of necrotic, cystic, vascular, or perinodal structures, the ROI size and placement may influence the ADC values and may not fully represent intranodal diffusion heterogeneity. Previous studies have shown that the ROI size can affect the ADC measurements in cervical lymph nodes [15,16].

Because no upper size threshold was applied, the cohort included both borderline-sized lymph nodes and clearly enlarged or morphologically suspicious nodes. This reflects routine clinical practice but may have increased the apparent diagnostic performance of morphological and ADC-based criteria. Therefore, the results should not be interpreted as being specific to borderline 7–10 mm lymph nodes.

Histopathological heterogeneity represents an important consideration when interpreting these findings. The cohort included malignant lymph nodes from different tumor types, including squamous cell carcinoma, thyroid carcinoma, lymphoma, and salivary gland malignancy, as well as benign and inflammatory entities such as reactive lymphadenopathy, CMV lymphadenitis, tuberculous lymphadenitis, and cystic lymphangioma. This heterogeneity reflects the real-world spectrum of cervical lymphadenopathy encountered in clinical practice, but it may also introduce biological variability in MRI appearance and ADC values. For example, metastatic carcinoma, lymphoma, granulomatous inflammation, and cystic benign lesions may demonstrate different diffusion profiles and enhancement patterns [12,14]. Therefore, the reported diagnostic performance should be interpreted as applying to a mixed cohort of cervical lymphadenopathies rather than to a single histopathological entity.

From a clinical perspective, these results indicate that nodal evaluation should not rely solely on size criteria or single imaging parameters. Instead, the integration of morphological features and diffusion metrics may enhance diagnostic confidence, improve patient stratification, and optimize biopsy selection. Such an approach aligns with contemporary imaging paradigms that favor multiparametric evaluation over isolated biomarkers.

Morphological criteria remain the foundation of cervical lymph node evaluation. Prior studies comparing traumatic neuroma with recurrent lymphadenopathy after neck dissection demonstrated that the short-axis diameter, short-to-long axis ratio, and specific T2-weighted signal characteristics significantly aid differential diagnosis, with structural parameters often outperforming other imaging features [17]. These findings reinforce our observation that nodal shape and margin characteristics are independent predictors of malignancy. Although the axial long-to-short ratio was significant in the univariate analysis, its loss of significance in the multivariate modeling suggests that comprehensive morphological assessment may better capture nodal architectural distortion than isolated two-dimensional measurements.

The diagnostic contribution of diffusion-weighted imaging has been consistently supported in several anatomical regions. Studies evaluating DWI and diffusion tensor imaging (DTI) in mediastinal lymphadenopathy reported significantly lower ADC values in malignant nodes, with AUC values between 0.89 and 0.96 [18,19,20]. Similarly, a meta-analysis of cervical lymphadenopathy confirmed significant weighted mean ADC differences between malignant and benign nodes [21]. In line with these reports, malignant lymph nodes in our cohort demonstrated significantly lower ADC values, with very good standalone diagnostic performance (AUC = 0.886). Importantly, however, the integration of the ADC with morphological parameters resulted in a substantial increase in the discriminative accuracy (AUC = 0.965), highlighting the incremental value of quantitative diffusion metrics within a multiparametric framework.

The diagnostic contribution of DWI may vary according to the anatomical region, tumor type, nodal size, and disease burden. In non-cervical nodal stations, including pelvic lymph nodes in gynecologic malignancies, previous studies have reported a more limited incremental value of DWI over conventional size-based criteria [22,23]. These discrepancies likely reflect differences in the lesion size, the prevalence of microscopic metastatic deposits, and regional imaging constraints. In contrast, the present cohort focused on clinically palpable cervical lymphadenopathy, where structural distortion and increased cellularity were more pronounced, allowing ADC to demonstrate robust discriminative performance.

Reactive lymphadenopathy has been reported to preserve normal diffusion characteristics in several inflammatory contexts. Longitudinal MRI studies of COVID-19 vaccine-related axillary lymphadenopathy showed a regression in nodal size and T2 signal over time, while the ADC values remained stable and within normal limits [24]. Such findings suggest that uncomplicated reactive hyperplasia does not typically produce significant diffusion restriction, supporting the biological plausibility of ADC as a marker of increased cellular density.

Nevertheless, diffusion restriction is not specific to malignancy. In our cohort, a histopathologically confirmed case of tuberculous lymphadenitis demonstrated low ADC values, rounded morphology, and heterogeneous enhancement, closely mimicking malignant involvement. Previous studies evaluating mediastinal tuberculous lymph nodes have similarly shown that ADC values may remain relatively stable during treatment, despite morphological changes [25,26]. These observations indicate that diffusion restriction in tuberculosis reflects granulomatous tissue composition and caseating necrosis rather than malignancy per se, underscoring the importance of integrating the clinical and morphological contexts to avoid false-positive interpretation.

In contrast, cystic lymphangioma exhibited imaging characteristics consistent with a benign fluid-containing lesion, including marked T2/STIR hyperintensity, minimal peripheral enhancement, and the absence of diffusion restriction. These features align with previously described MRI findings of lymphangiomas, which typically demonstrate pure fluid signal intensity with thin septations and no solid enhancing components [27,28,29]. MRI has been shown to be particularly effective in the noninvasive characterization of cystic neck lesions, allowing differentiation from malignant masses based on signal profile and enhancement pattern [30]. In our case, preserved diffusivity combined with characteristic morphology strongly supported a benign diagnosis.

Artificial intelligence and radiomics-based approaches represent a promising future direction for improving MRI-based assessment of cervical lymphadenopathy. Unlike conventional visual analysis, radiomics can extract high-dimensional quantitative features related to shape, intensity, texture, and diffusion heterogeneity, which may capture subtle intranodal changes not readily apparent to the human observer. Recent studies and systematic reviews suggest that radiomics and machine learning models may improve the prediction of cervical lymph node metastasis in head-and-neck cancer, particularly when multiparametric MRI features are combined with ADC values and conventional nodal characteristics. However, these methods remain limited by their segmentation variability, small single-center datasets, heterogeneous imaging protocols, and insufficient external validation [31,32,33]. Therefore, AI-based approaches should currently be regarded as complementary research tools rather than replacements for structured radiological assessment, with future multicenter studies needed to confirm their clinical utility.

Several limitations should be acknowledged. First, the study was conducted on a relatively limited unicentric sample size, which may increase the risk of model overfitting. Second, the DWI protocol was not fully homogeneous across all cases, as high-b-value diffusion weighting varied between b = 800 and b = 1000 s/mm^2^, with b = 50 s/mm^2^ serving as the low-b-value reference rather than a true b = 0 acquisition. Although the ADC values did not differ significantly between protocols in the sensitivity analysis, this heterogeneity may limit measurement comparability and should be addressed in future prospective studies using standardized DWI acquisition. Another limitation is the absence of complete blinding during retrospective image analysis. Although most original clinical MRI reports were generated before histopathological confirmation, the research team performing the retrospective data extraction had access to imaging reports and pathological results. This may have introduced observer bias, particularly for qualitative morphological features. Future prospective studies should incorporate fully blinded independent readers. The sample size remains limited, and the findings should be considered exploratory until externally validated in larger multicenter cohorts. In addition, although lymph nodes smaller than 7 mm were excluded to improve the measurement reliability, the slice thickness varied between 3 and 5 mm, depending on the sequence and imaging plane. A 5 mm slice thickness may still introduce partial-volume effects, particularly for smaller lymph nodes near the lower inclusion threshold. This represents an additional limitation and supports the need for prospective studies using standardized high-resolution MRI protocols.

The histopathological heterogeneity of the cohort represents a major limitation. Although this reflects real-world clinical practice, the inclusion of multiple malignant and benign etiologies may limit the generalizability of the findings to specific disease entities. Because several histopathological subgroups contained only a small number of cases, diagnosis-specific subgroup analysis was not feasible. Consequently, the model should be interpreted as a tool for binary benign-versus-malignant stratification in a mixed cervical lymphadenopathy cohort, rather than as a histology-specific classifier, although larger multicenter studies with more homogeneous disease-specific subgroups are required to validate these findings across individual histopathological categories.

## 5. Conclusions

The present study suggests that ADC values, nodal shape, and margin characteristics provide complementary information for differentiating benign from malignant cervical lymph nodes on MRI. Within the methodological framework of the present study, ADC alone demonstrated good diagnostic performance, while its combination with qualitative morphological features improved discrimination in a lesion-based model accounting for intra-patient clustering.

Therefore, a structured multiparametric MRI assessment may support cervical lymph node stratification and help to identify nodes that warrant histopathological confirmation or closer follow-up. However, given the retrospective single-center design, limited sample size, heterogeneous histopathological spectrum, and absence of external validation, these findings should be interpreted as exploratory and require confirmation in larger prospective multicenter cohorts.

## Figures and Tables

**Table 1:** Histopathological and clinicopathological breakdown of the 88 lymph nodes included in the analysis.

Histopathological/ Clinicopathological Category	Patients	Lymph Nodes	Malignant Nodes	Benign Nodes
Reactive/ inflammatory lymphadenopathy	23	45	0	45
Squamous cell carcinoma	13	24	24	0
Papillary thyroid carcinoma	2	4	4	0
Reactive/benign lymphadenopathy, unspecified	1	4	0	4
Non-Hodgkin lymphoma	1	3	3	0
CMV lymphadenitis	1	3	0	3
Reactive nodes associated with Warthin tumor	1	1	0	1
Adenoid cystic carcinoma	1	1	1	0
Tuberculous lymphadenitis	1	1	0	1
Medullary thyroid carcinoma	1	1	1	0
Cystic lymphangioma	1	1	0	1
Total	39 *	88	33	55

* Some patients contributed to more than one category; therefore, category-level patient counts are not additive.

**Table 2:** Association between MRI morphological features and malignancy.

Feature	Malign n/N (%)	Benign n/N (%)	OR	95% CI	*p*-Value
Round shape	23/33 (69.7)	6/55 (10.9)	18.2	6.0–54.6	<0.001
Lobulated/irregular margins	14/33 (42.4)	2/55 (3.6)	19.5	4.0–94.0	<0.001
Necrosis present	6/33 (18.2)	1/55 (1.8)	12.0	1.3–104.7	0.006
Preserved fatty hilum	0/33 (0.0)	51/55 (92.7)	0.001	0.00007–0.026 *	<0.001

* OR and 95% CI for the preserved fatty hilum were estimated using Firth’s penalized logistic regression, because conventional logistic regression showed complete separation.

## Data Availability

All data are available from the corresponding author/Supplementary Materials upon reasonable request due to privacy reasons.

## Supplementary Materials

The following supporting information can be downloaded at: https://www.mdpi.com/article/10.3390/healthcare14111524/s1, STARD 2015 for the manuscript Multiparametric MRI Assessment of Cervical Lymphadenopathy: Combined Diagnostic Performance of Morphological Features and Apparent Diffusion Coefficient by Iulian-Alexandru Taciuc, Mihai Dumitru, Daniela Vrinceanu, Andreea Nicoleta Marinescu, Crenguta Serboiu, Adrian Costache and Adina Zamfir-Chiru-Anton.

## Abbreviations

The following abbreviations are used in this manuscript:

ADC	apparent diffusion coefficient
AUC	area under the curve
CI	confidence interval
CT	computed tomography
DTI	diffusion tensor imaging
DWI	diffusion-weighted imaging
ENE	extranodal extension
EPI	echo-planar imaging
ENT	ear, nose and throat
L/l	long-to-short axis diameter ratio (axial)
MRI	magnetic resonance imaging
OR	odds ratio
PACS	picture archiving and communication system
ROC	receiver operating characteristic
ROI	region of interest
STIR	short tau inversion recovery
US	ultrasonography
VIF	variance inflation factor
